# Genetic variation for resistance to high temperature stress of mature sperm – a study in Drosophila

**DOI:** 10.1371/journal.pone.0173990

**Published:** 2017-03-30

**Authors:** Jørgen Bundgaard, J. S. F. Barker

**Affiliations:** 1 Section for Genetics, Ecology and Evolution, Department of Bioscience, Aarhus University, Ny Munkegade 116, Aarhus, Denmark; 2 School of Environmental and Rural Science, University of New England, Armidale, NSW, Australia; National Cancer Institute, UNITED STATES

## Abstract

Genetic variation for resistance to heat stress has been found for a number of life-history components in Drosophila species. For male and female fertility (or sterility), stress resistance of the parents is confounded with stress resistance of the haploid gametes. Many genes are known to influence male fertility in *Drosophila melanogaster*. Some may carry temperature sensitive alleles that reduce fertility through effects on mature sperm when exposed to heat stress. In this study, sperm from each of 320 males were either not heat shocked (control) or exposed to a heat shock (36.9°C for 2 hours) either in the male testes or in the female reproductive tract. We did not detect any temperature sensitive sterility alleles. These results are relevant in relation to haploid gene expression and the findings of considerable amounts of mRNA in mature sperm, potentially important for sperm function and fertilization.

## Introduction

There is ample evidence that genetic variation for resistance to heat stress can be a significant factor in determining species distributions, and global warming is likely to move species borders towards higher latitudes and altitudes, unless the species is able to adapt to the warmer climate [1,2,3]. In species of Drosophila, genetic variation for heat resistance has been demonstrated for a number of life-cycle components such as egg hatchability [4], egg-to-adult survival [5], pupal survival [4,6], female fecundity [7], male fertility and male sterility [8,9,10,11], sperm motility [12] and male mating ability [10,13].

All of the above life-cycle components deal with the diploid stages, except for male and female sterility and sperm motility, where stress resistance of the parents is confounded with resistance of the haploid gametes. There is good evidence that many genes can influence male fertility in *Drosophila*. An extensive screen [14] of 2131 independent lines carrying male sterile mutations on the 2^nd^ and 3^rd^ chromosomes of *Drosophila melanogaster*, which constitute 80% of the genome, found that more than 400 genes could mutate to male sterile mutations. The lines were classified cytologically, and 19% (or 404) of the mutations affected mature sperm, including the fertilization process. How many of these genes could be expected to carry temperature sensitive alleles with lethal, sublethal or smaller fitness effects is not known. It has been shown [5,15] that approximately 10% of all lethals seem to be temperature sensitive (conditional) lethals at 29–30°C in *D*. *melanogaster*. The frequency of conditional sterility alleles is not known. However, it has been calculated [16] that at least 250 genes could mutate to male sterile alleles, but the number of genes required for fertility in either sex was likely to be much greater. Further, many mutations affecting a wide variety of genes have pleiotropic effects on the male germ line leading to male sterility [17]. It is possible then that the 2^nd^ and 3^rd^ chromosomes from the G83 population used in this study harbor a number of genes that could carry temperature sensitive alleles influencing survival and function of mature sperm after heat stress.

The objective of this study was to investigate the frequencies and effects of these conditional sterile alleles in a large random mating population of *Drosophila melanogaster*. To our knowledge it is the first study of this kind in any organism.

## Materials and methods

### Population and stocks

The *Drosophila melanogaster* population G83 was chosen as the base population for these experiments. This population was founded in 1983 from 403 females captured at the local fruit market in Groningen, The Netherlands, and since maintained as a large population at ca. 25°C [18]. Hence, the population could be expected to have maintained a high degree of genetic variability and due to the constant temperature at 25°C, possible heat sensitive alleles could be expected to occur at least at mutation-selection equilibrium. That this large population is still genetically variable for fitness genes has been shown by a number of recent studies [5,11,19].

The translocated balancer stock T(2;3)CyO-TM6, CyO:TM6/ry^506^Sb^1^P{Δ2–3}99B (named Cy in the following after the dominant wing character Curly) was used to extract 2^nd^ and 3^rd^ chromosomes simultaneously from the G83 population. This stock (no.106065) and the Oregon-R wild type stock used in the experiments were both procured from the Bloomington Stock Center at Indiana University, USA.

### Preliminary experiments

The aim of the main experiments was to determine if heat stress of mature sperm exposed genes carrying alleles with conditional effects on sperm survival, fertilization and offspring viability. Thus, we needed to identify combinations of stress temperature and exposure time that severely stressed the flies (and sperm) without killing most of the flies. Sperm from males that were heterozygous for the balancer chromosomes carrying Cy, and the wild type 2^nd^ and 3^rd^ chromosomes (see Experimental procedure below) were exposed in both males and mated females in water baths to a number of temperatures between 36.0±0.1°C and 38.0±0.1°C for 30 min to 3 hrs. Heterozygous Cy/++ males and wild type Oregon-R females inseminated by these heterozygous males were treated and both the segregation ratio between the balancer chromosome and wild type and the number of surviving offspring were recorded (data not shown). On the basis of these preliminary experiments we decided to expose the flies to 36.9 ±0.1°C for two hours in the experiments described below. This stress temperature reduced the number of producing females (i.e. surviving and with live sperm) by some 50%, and the number of progeny was reduced as compared with a milder stress. Higher stress temperatures and longer exposures gave too low survival.

### Experimental procedure

**Fig 1:**
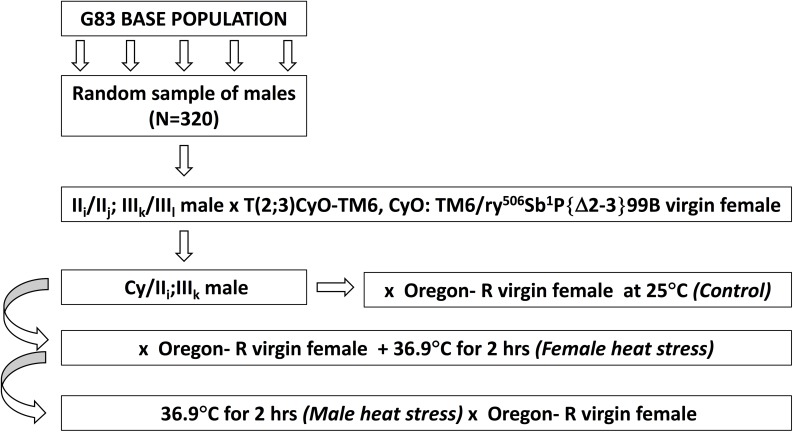
Diagrammatic representation of the experimental procedures.

A random sample of 320 males from the G83 population was crossed individually to females from the Cy-labelled balancer stock and from each cross one Curly F_1_ male (i. e. Cy/II_i_;III_k_) was used for further experimentation (Fig 1).

Due to the reciprocal translocation, the expected result from meiosis in the male would be sperm with the translocated chromosomes (II + III) together carrying the Cy allele on the second chromosome, or the extracted chromosomes II and III together being wild type for the Curly locus. No other viable chromosome combinations were expected in the progeny. The segregation ratios in the progeny of these heterozygous males are studied in three situations: 1) Control, 2) Female heat stress, and 3) Male heat stress. Each male was used in all three treatments such that his mature sperm were studied in 1) an unstressed environment, 2) when heat stressed inside the inseminated female, and 3) when heat stressed in the male himself. Ten males, of the 320 tested, failed to produce progeny in any of the three treatments.

#### Control

In the Control treatment at 25°C, two-day old virgin Cy/++ males were each mated to one two-day old Oregon-R virgin female for 24 hours in numbered vials. The males were then transferred to the *Female heat stress* treatment, while the mated females were allowed to lay eggs in the vial for 3 days. When all the progeny emerged, thirty were randomly chosen (or all progeny if less than 30), phenotypically scored as Curly or wild type, and the segregation ratio Cy: wild type recorded for each vial.

#### Female heat stress

All heterozygous Cy/++ males from the Control treatment were mated individually to a fresh two-day old virgin Oregon-R female for 24 hours (in numbered vials, same numbers as in the Control). The male and female (in the same vial) were then exposed to 36.9 ±0.1°C for 2 hrs. in a water bath. After treatment, the females were placed individually in fresh, numbered vials where they laid eggs for a total of nine days (transfers to fresh vials every three days). The males were transferred to fresh, numbered vials with virgin Oregon-R females (Male heat stress treatment, see below).

#### Male heat stress

As mentioned above, the heterozygous males from the *Female stress* treatment were, after exposure, mated individually to one two-day old virgin Oregon-R female for 24 hrs. The male then was discarded and the female allowed to lay eggs for nine days (transferred to fresh vials every three days).

In both stress treatments, when all progeny emerged, thirty were randomly chosen from each of the three vials (or all progeny if less than 30), phenotypically scored and the segregation ratio Cy: wild type recorded.

If temperature sensitive alleles are present in the G83 population, and are expressed by the experimental heat treatment, then the proportion of ++ progeny is expected to be lower in the stress treatments than in the Control. In the three experiments, we identified and counted a total of 27,174 flies.

## Results

In order to ensure that any treatment effects are correctly assessed, we have done one-way ANOVA of Control vs. Female stress, Control vs. Male stress and Female stress vs Male stress for: (a) All males in each treatment, (b) All males in each treatment, but first vial only progeny, (c) Males that produced progeny on all three treatments, (d) Males that produced progeny in both Control and Female stress, (e) Males that produced progeny in both Control and Female stress, but first vial only progeny, (f) Males that produced progeny in both Control and Male stress, (g) Males that produced progeny in both Control and Male stress, but first vial only progeny, (h) Males that produced progeny in both Female and Male stress, (i) Males that produced progeny in both Female and Male stress, but first vial only progeny.

**Fig 2:**
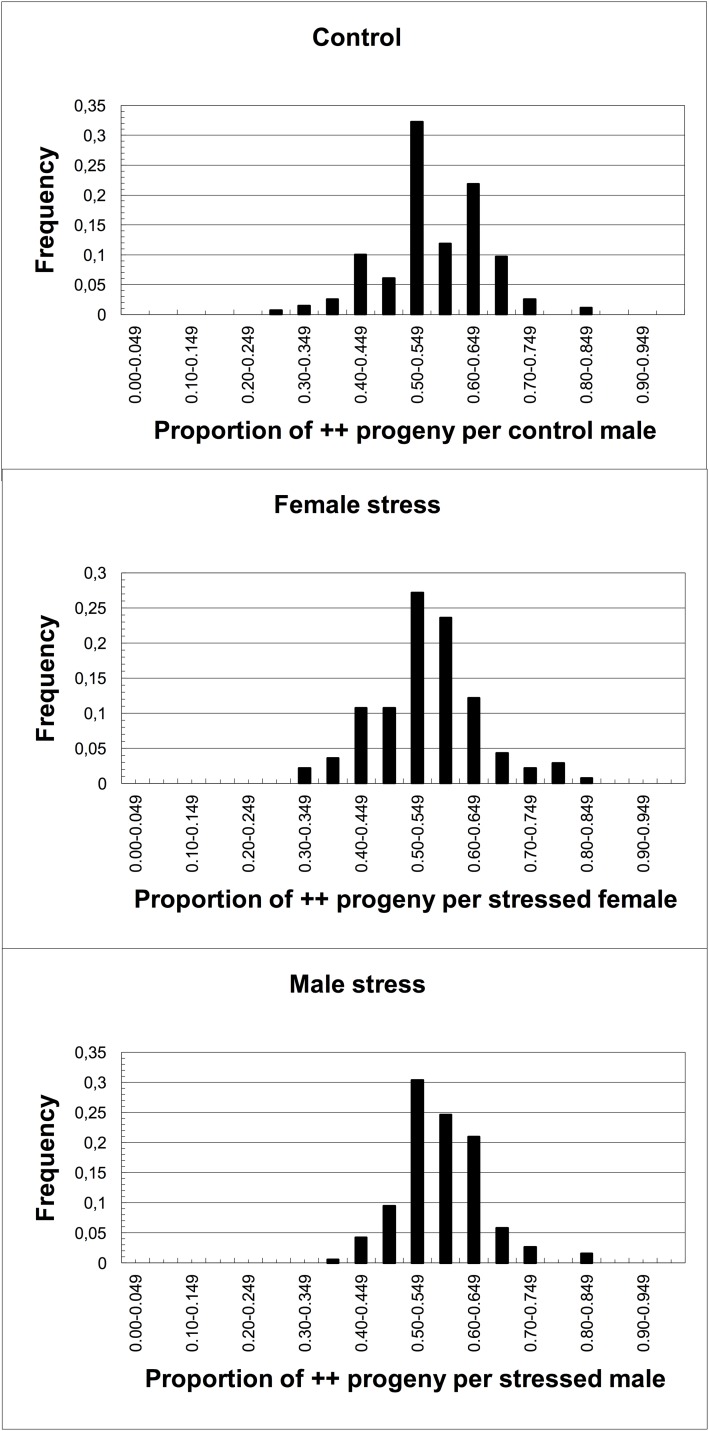
Frequency distribution of the proportion of ++ progeny for each treatment.

The numbers of males that produced progeny, the mean numbers of progeny scored per male, the mean proportion of ++ flies in the progeny and the proportion of sterile matings are given in Table 1, and the frequency distributions of the proportion of ++ progeny in Fig 2.

**Table 1:** Number of males that produced progeny, mean number (±SD) of progeny per male and mean proportion (±SD) of ++ progeny in each treatment comparison.

**(a) all males in each treatment**			
**Treatment**	**Control**	**Female stress**	**Male stress**
Number of males that produced progeny	279	144	194
Mean number of progeny/male	29.53±2.78	44.83±19.00	64.29±20.73
Mean proportion ++ progeny	0.545±0.093	0.542±0.099	0.563±0.076
Proportion sterile	0.1	0.535	0.374
**(b) all males in each treatment, but progeny in first transfer vial only**			
Number of males that produced progeny	279	140	190
Mean number of progeny/male	29.53±2.78	22.84±8.81	28.41±5.01
Mean proportion ++ progeny	0.545±0.093	0.551±0.152	0.559±0.103
Proportion sterile	0.1	0.548	0.387
**(c) males that produced progeny in all three treatments**			
Number of males that produced progeny	89	89	89
Mean number of progeny/male	29.80±0.10	43.79±18.69	63.35±20.90
Mean proportion ++ progeny	0.548±0.101	0.538±0.094	0.561±0.081
**(d) males that produced progeny in both the control and female stress treatments**			
**Treatment**	**Control**	**Female stress**	**Male stress**
Number of males that produced progeny	125	125	
Mean number of progeny/male	29.65±2.27	43.94±18.90	
Mean proportion ++ progeny	0.550±0.092	0.540±0.101	
**(e) males that produced progeny in both the control and female stress treatments–first vial only**			
Number of males that produced progeny	123	123	
Mean number of progeny/male	29.64±2.29	22.36±9.08	
Mean proportion ++ progeny	0.551±0.093	0.542±0.101	
**(f) males that produced progeny in both the control and male stress treatments**			
Number of males that produced progeny	176		176
Mean number of progeny/male	29.61±2.66		64.03±20.78
Mean proportion ++ progeny	0.541±0.097		0.565±0.077
**(g) males that produced progeny in both the control and male stress treatments—first vial only**			
**Treatment**	**Control**	**Female stress**	**Male stress**
Number of males that produced progeny	172		172
Mean number of progeny/male	29.70±2.33		28.40±4.86
Mean proportion ++ progeny	0.543±0.096		0.565±0.077
**(h) males that produced progeny in both the female stress and male stress treatments**			
Number of males that produced progeny		100	100
Mean number of progeny/male		43.65±18.20	63.70±20.05
Mean proportion ++ progeny		0.539±0.094	0.560±0.079
**(i) males that produced progeny in both the female stress and male stress treatments- first vial only**			
Number of males that produced progeny		94	94
Mean number of progeny/male		23.49±8.52	28.56±4.67
Mean proportion ++ progeny		0.540±0.094	0.562±0.079

As each male was exposed to all three treatments, the smaller numbers of offspring in the two stress treatments (clearly shown in the first vial only comparisons, (b), (e) and (g)), are due to mortality and sterility induced by the heat stress [4,20]. Of all the ANOVAs that were performed comparing the mean proportions of ++ progeny, only four were significant:

1. All males in each treatment, Control vs Male (a), F_(1,471)_ = 4.79, P = 0.03,
2. All males in each treatment, Female vs Male (a), F_(1,336)_ = 4.72, P = 0.03,
3. Males that produced progeny in both Control and Male stress (f), F_(1,350)_ = 6.73, P = 0.01.
4. Males that produced progeny in both Control and Male stress, but first vial only (g), F_(1,342)_ = 5.53, P = 0.02.

Where variances were significantly different, tests of treatment effects assuming equal or unequal variances gave similar results, and where appropriate, weighted ANOVA allowing for variation in the number of progeny scored per male gave similar results.

## Discussion

In the Male stress treatment, the sperm transferred to females had been subjected to the heat stress 24–48 hours previously. Thus at least some of the sperm transferred would have been in the later stages of sperm differentiation at the time of exposure to the heat stress. In contrast, in the Female stress treatment, only mature sperm would have been transferred to the females. In either case, effects of heat stress will be detected only if temperature sensitive sterile alleles are present in the G83 population and are expressed during the heat shock treatment.

If temperature sensitive alleles in the G83 population are exposed by the heat stress, the proportion of ++ progeny from sperm heat stressed in males or in females will be reduced as compared with Control males. This would result both in a skew segregation ratio (Table 1) and a left tail in Fig 2. Note that this assumes there are no, or at least a lower frequency of, temperature sensitive sterile alleles on the balancer chromosomes. If there are temperature sensitive alleles on the balancer chromosomes, and none on the tested wild chromosomes, then the proportion of ++ progeny should be greater than 0.5 in both treatments, and significantly greater than in the Control treatment.

The percentage of genes expressed in male gametes is known to be high in plants while it is considered to be low in animals. In plants, some 12%-65% of the genes are expressed in pollen, while in mice and rats only 1.3%-3.8% are expressed in sperm. The latter estimates still amount to hundreds to thousands of genes in these animals [21].

*de novo* RNA transcription at post-meiotic phases of spermatogenesis has been observed [22], and these authors suggested that “a subset of these RNAs is packaged into mature sperm and delivered to the egg at fertilization”. This was confirmed [23], by showing that mature sperm in *D*. *melanogaster* deliver mRNA transcripts to the egg during fertilization, and that these were unlikely to be derived from contaminating somatic cells or immature sperm. Such mRNA transcripts have also been demonstrated in man, mouse and horse sperm [24,25]. In human males, a subset of sperm RNAs is present in altered amount in infertile patients [26]. For three of these transcripts, this differential expression has been confirmed, and a positive correlation between expression and sperm motility shown [27]. Subsequently, a set of sperm RNAs whose absence correlates with infertility has been identified [28]. Further, protein translation has been shown to occur in mammalian sperm during their residence in the female reproductive tract until fertilization [29]. If these findings are true also for *D*. *melanogaster*, and if conditional sterile alleles are present in the G83 population, and among those that are expressed, then such temperature sensitive sterility alleles could be detected in both treatments.

Comparison of the distributions for the Control and Female stress and for the Control and Male stress (Fig 2) shows no sign of conditional sterile alleles, which would have resulted in a left tail in the Female and Male stress plots. In fact, for those comparisons that were significant, the results are opposite to that predicted, viz. the proportion of ++ progeny in the Male stress treatment being greater than in the Control. This could be due to a generally lower survival of the Cy/++ flies whose Cy-labelled chromosomes carry a number of mutant alleles. In contrast, sperm stressed in the female reproductive tract do not show this effect. As none of the Control vs Female stress comparisons were significant, we have not detected temperature sensitive alleles in either the G83 population or in the balancer chromosomes.

Our results indicate:

1. that temperature sensitive sterility alleles are not present in either the G83 population or balancer chromosomes, or
2. that if they are present, they are not expressed in spermatids or in mature sperm, or
3. that the treatments were not sufficiently intense.

Clearly our negative result is not definitive, and other populations and stress levels need to be investigated, particularly given the current intense study of fertility/sterility in all its many aspects.

## Supporting information

S1 Table Sperm sensitivity to heat stress. (XLS)
